# Why don't horseflies land on zebras?

**DOI:** 10.1242/jeb.244778

**Published:** 2023-02-17

**Authors:** Tim Caro, Eva Fogg, Tamasin Stephens-Collins, Matteo Santon, Martin J. How

**Affiliations:** School of Biological Sciences, 24 Tyndall Avenue, University of Bristol, Bristol BS8 1TQ, UK

**Keywords:** Aliasing, Domestic horses, Tabanids, Polarization, Stripes

## Abstract

Stripes deter horseflies (tabanids) from landing on zebras and, while several mechanisms have been proposed, these hypotheses have yet to be tested satisfactorily. Here, we investigated three possible visual mechanisms that could impede successful tabanid landings (aliasing, contrast and polarization) but additionally explored pattern element size employing video footage of horseflies around differently patterned coats placed on domestic horses. We found that horseflies are averse to landing on highly but not on lightly contrasting stripes printed on horse coats. We could find no evidence for horseflies being attracted to coats that better reflected polarized light. Horseflies were somewhat less attracted to regular than to irregular check patterns, but this effect was not large enough to support the hypothesis of disrupting optic flow through aliasing. More likely it is due to attraction towards larger dark patches present in the irregular check patterns, an idea bolstered by comparing landings to the size of dark patterns present on the different coats. Our working hypothesis for the principal anti-parasite features of zebra pelage are that their stripes are sharply outlined and thin because these features specifically eliminate the occurrence of large monochrome dark patches that are highly attractive to horseflies at close distances.

## Teaser

**Summary:** Pelage contrast and small dark patches are key to enabling stripes preventing zebras being attacked by biting flies.

## INTRODUCTION

Over the past decade, evidence for zebra striping being an adaptation to thwart biting fly attack has continued to grow, while suggestions that stripes confuse predators, are a form of camouflage or are a thermoregulatory mechanism all lack empirical support (Caro, 2020). In brief, stripes reduce landings of tabanid horseflies based on experimental studies with striped artificial targets (Brady and Shereni, 1988; Gibson, 1992; Sasaki et al., 2020; Waage, 1981), horse models (Egri et al., 2012a), human models (Horváth et al., 2019), painted cows (Kojima et al., 2019) and comparisons of live plains zebras (*Equus burchelli*) with domestic horses (Caro et al., 2019; How et al., 2020), and there is a co-occurrence of tabanid annoyance and striping in wild equids (Caro et al., 2014). In contrast, observational studies of zebras fleeing do not support a confusion effect (Caro, 2016), stripes can only be resolved by predators at close distances undermining camouflage ideas (Melin et al., 2016) and experiments with striped objects find no support for a cooling effect (Horváth et al., 2018). Nonetheless, the mechanism by which stripes deter biting flies from landing is still poorly understood and lack of knowledge of a mechanism can reduce the credibility of trait function.

Several studies of free-living biting flies, usually tabanids, have attempted to tease out mechanisms underlying the anti-parasite effect of striping (Caro et al., 2022). Commercial white, black and striped horse coats placed sequentially on domestic horses were found to generate a marked reduction in tabanid landings on the striped compared with other coats but no difference was found in landing rates on the naked neck and heads (Caro et al., 2019). In separate, detailed video analyses, tabanids approached zebras faster and failed to decelerate before contacting zebras, and proportionately more tabanids simply touched rather than landed on zebra pelage in comparison to horses at the same livery. Taken together, these findings indicate that horseflies are attracted to hosts from a distance but are prevented from appropriate landing behaviour close to their target (Caro et al., 2019).

How and colleagues (2020) investigated whether stripes might disrupt the radial symmetry of optic flow via the aperture effect (i.e. the generation of false motion cues by continuous straight edges). They recorded and reconstructed tabanid behaviour around horses wearing striped, checked and monochrome grey coats and found that flies avoided landing on, flew faster near and did not approach as close to striped and checked coats compared with grey. That flies avoided checked patterns in a similar way to stripes refutes the hypothesis that stripes disrupt radial optic flow via the aperture effect, which critically demands parallel striping patterns.

Female tabanids that need a blood meal to reproduce are strongly attracted to horizontally polarized light (Horváth, 2014; Horváth et al., 2017). Egri and colleagues (2012a) found that black stripes reflect light with a high degree of polarization (*d*>80%) whereas white stripes do not (*d*<5%). In a series of experiments involving sticky boards, painted trays filled with salad oil, and sticky half-sized horse models, Egri and colleagues (2012a,b) found that stripe width, polarization direction, brightness and degree of polarization may all affect host-seeking behaviour in tabanids. Conversely, Britten and co-workers (2016) argued that under field conditions, black and white stripes in wild plains zebras give off polarization signals that are similar in both degree and polarization angle, and that reflected polarized light is greatly influenced by the body area under consideration. Some of these discrepancies could be related to measurement issues (Tibbs et al., 2017) or to differences in lighting conditions.

Studies have also shown that the size of an object in the fly's visual field affects biting fly (tabanid, glossinid and *Stomoxys*) landings. In the laboratory, thin stripes are more aversive (Brady and Shereni, 1988; Egri et al., 2012a) and these correspond to the widths of stripes found on many parts of zebra pelage. Additionally, other patterns deter tabanids, including regular checkerboard patterns (How et al., 2020) as do dumbbell shapes regularly placed next to each other (Blaho et al., 2012), which are more effective the smaller they are in size; this result parallels the findings related to stripe thicknesses.

Taken together, these experimental and observational studies are difficult to interpret because they suggest that several mechanisms might be contributing to the efficacy of striping in preventing flies from making successful landings. Indeed, since zebras are black and white in coloration, have stripes that range from 1.21 to 3.90 cm in width taking plains, mountain (*Equus quagga*) and Grevy's zebra (*Equus grevyi*) species as a whole (Caro et al., 2014), stripes are spaced regularly, are highly contrasting, and may reflect polarized light differentially, it is possible that tabanids respond to several of these stimuli.

We therefore thought it productive to investigate these mechanisms further by examining the effects of (1) aliasing, (2) polarization, (3) contrast, and, inadvertently, (4) pattern size on tabanid behaviour, although the last exploration suffered from confounding variables.

Where regular patterns are viewed by the fly while in motion, they could become misregistered, producing false motion directions and magnitudes (How and Zanker, 2014), an example being the wagon-wheel effect for a human observer (How et al., 2020). This misperception of motion could work against the tendency for the fly to fixate the pattern, creating positive feedback and causing it to turn away, but crucially the mechanism depends on pattern regularity. We explored this by comparing the responses of free-living tabanids exposed to a regular checkerboard stimulus pattern versus a randomised checker pattern. If aliasing is in operation, the irregular randomized check pattern should fail to thwart landings.

Given that equid pelage reflects the polarization of light differentially according to region of the body (Britten et al., 2016; Egri et al., 2012a) being greater on the neck, dorsum and rump, especially when the light source illuminates the animal from behind, and because black surfaces reflect a higher degree of polarization than white surfaces (Egri et al., 2012a,b), we tested whether black patches placed dorsally were more effective than white patches, which reflect less polarized light.

Since one of the salient features of zebra pelage is highly contrasting black and white stripes, we wanted to understand the importance of differences in contrast between stripes for tabanids. We investigated this using a series of identically striped patterns varying only in the contrast between the light and dark stripes.

Finally, we had the fortuitous opportunity to extend understanding of patch size in affecting the behaviour of free-living tabanids, by sorting our results according to the size of monochrome dark patches across our experimental treatments and an additional control haphazard coat pattern. For all these experiments we used domestic horses on which we placed differently patterned coats as utilized successfully in previous studies (Caro et al., 2019; How et al., 2020).

## MATERIALS AND METHODS

### Coat design

**Fig. 1.**
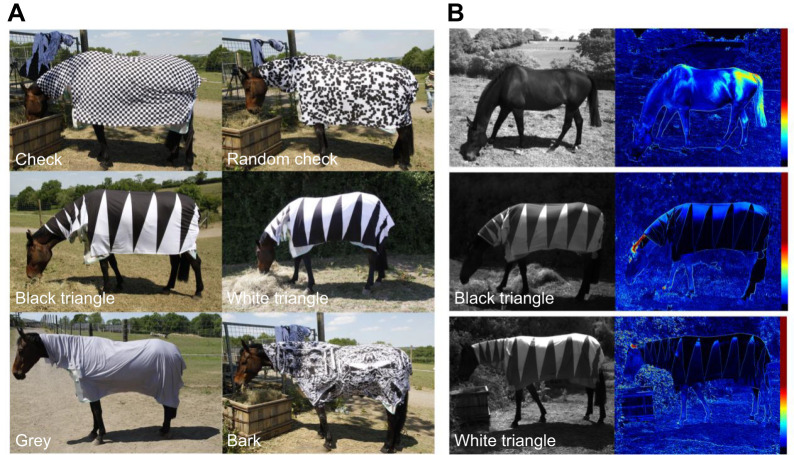
**Horse coats with patterns photographed *in situ*.** (A) Colour photographs of the check, random check, black triangle, white triangle, grey and bark coat patterns. (B) Polarization images of a bare horse (top), and black triangle (middle) and white triangle (bottom) coats. Images on the left are monochrome photographs viewed through horizontally oriented polarizing filter. Images on the right are false-coloured according to the right-most scale to represent degree of polarization.

Rolls of fabric (Single Jersey cotton, 180gsm) were custom printed (CottonBeeUK, London, UK) with 11 patterns as follows: (i–v) 3.5 cm width vertical stripes of four differing contrasts as well as a uniform grey control. The relative reflectance of stripes on each coat was measured using a spectrophotometer (OceanHDX, Ocean Insight, Florida, USA) coupled to a bifurcated fibre-optic probe and illuminated using a DH2000 Halogen light source (Ocean Insight). Reflected intensity in the 400–700 nm range was used to calculate the Michelson contrast; (vi) a 3.5 cm width regular black and white checkerboard; (vii) a 3.5 cm width irregular black and white checkerboard generated by randomly placing black squares on the white background until 50% of the total area was covered; (viii) a series of very large right-angle black triangles (100×33 cm) below which were white triangles; (ix) a series of very large white triangles (100×33 cm) below which were black triangles; (x) a uniform grey control, (2.2 m^2^ when viewed from one side); and (xi) a naturalistic ‘bark’ pattern produced by applying a 50:50 binary threshold to a monochrome photograph of understory twigs and leaf litter (Fig. 1A). Print shades were chosen so that all patterns, had approximately the same average luminance. This was achieved by ensuring that patterned rugs were printed with a 1:1 ratio of black and white areas, and the mid-grey shade (relative luminance=0.46) closely matched the mean relative luminance of black (0.075) and white (1) fabric. Patterned coats were assembled by sewing together the printed fabric to make a 2.0×2.0 m square covering the horse's body and a smaller 0.8×0.5 m rectangle covering the neck and withers. We estimated the size of the dark patterns from the visual perspective of horseflies at 1 m distance from targets by filtering out all dark patches smaller than a visual angle of 1 deg (2.4 cm^2^). Dark patch size was then estimated as half the rug's area for the grey coat (20,000 cm^2^), the area of a triangle for black and white triangle coats (1666.7 cm^2^), the 95th percentile of dark patch sizes for bark (980.6 cm^2^) and for irregular checkerboard (194.6 cm^2^) and the size of a check for the regular checkerboard (12.25 cm^2^). For comparison, a single stripe on the contrasting stripe rugs had a total area of 700 cm^2^. The polarization properties of a naked horse and horses wearing the two triangle coats (viii and ix) were measured using a polarization camera (Triton, Lucid Vision Labs Ltd, Richmond, Canada) and displayed as heat maps indicating the degree of polarization (Fig. 1B). To avoid the problem of false polarization measurements induced by sensor noise in dark regions of the image (Tibbs et al., 2017) pixels in the lowest 5% range of intensity values were removed from the analysis.

### Experimental protocol

The five striped coats of differing Michelson contrast (0, 0.124, 0.330, 0.595, 0.8641) were placed in random order on 10 different horses sequentially for approximately 10 min each and affixed to underlying fly coats with safety pins. In a separate experiment the six patterned coats were placed in random order on 16 horses. Both experiments occurred between 25 June and 22 July 2021 at four horse liveries in Lansdown, Abbots Leigh and Burrington, North Somerset, UK, and Okeford Fitzpaine, Dorset, UK. All work was assessed and approved by the university Animal Welfare and Ethical Review Body (AWER) at the University of Bristol (UIN number UB/18/074).

### Recording of fly landings

One observer filmed flies approaching on one side of the horse. Usually, several different flies landed on horses each observation period, with repeat landings by single horseflies being documented from the flight trajectories. Filming was conducted using digital video cameras (Hero 5 or 6, GoPro, San Mateo, USA) affixed at either end of a horizontal 1.0 m metal bar mounted on a tripod. The cameras were positioned to approximately maintain their horizontal and vertical axes relative to the outside world and provided two different viewing angles on the horse to aid subsequent analysis. Fly activity around horses was filmed at 60 frames s^−1^, at a resolution of 2704×1520 pixels. Horsefly (*Haematopota pluvialis* and *Tabanus bromius*) landings were manually extracted from pairs of video recordings by two independent observers (E.F., T.S.-C.), first by identifying tabanids approaching the horse, then observing their landings. We separated the data by whether all landings occurred only on the coat, all landings on the edge of the coat, or on the naked head, legs and belly (combined). Fly trajectories observed when the horse was ambulating were discarded and all landings on the immediate trailing edge of the coat were subsequently excluded from the analyses due to uncertainty over the visual experience of flies during the final moments of approach.

### Statistical analyses

Videos from one horse wearing a grey rug were excluded from the analyses because of poor lighting conditions that precluded reliable scoring. To investigate the effects of aliasing (experiment 1), polarization (experiment 2), contrast (experiment 3) and pattern size (experiment 4) on tabanid behaviour, we implemented generalised linear mixed models in R v. 4.1.2 (https://www.r-project.org/) using the brms package (Bürkner, 2018), which fits Bayesian models using Stan (https://mc-stan.org/). The first model (*N*=16) analysed the number of horsefly landings between horses wearing different patterned rugs (testing experiments 1 and 2) using a Poisson distribution with log-link. The model included the main predictors ‘coat type’ (grey, regular checkerboard, irregular checkerboard, black triangles, white triangles), ‘landing area’ (coat, naked skin), ‘scorer of the videos’ (E.F, T.S.-C.) and their interactions. To express landings in relation to the underlying observation duration, we further included the log-transformed offset term ‘video duration’ (minutes). To account for the repeated measurements on each horse we also included ‘horse ID’ as random intercept to the model. The model further included random slopes over ‘coat type’ and ‘landing area’ because their relationship with number of horsefly landings varied among horses.

The second model (*N*=10) tested the contrast hypothesis (experiment 3) by comparing the number of horsefly landings between horses wearing striped coats using a negative binomial distribution with log-link. The main difference from the previous model was that the main predictor ‘coat type’ was now replaced by the continuous covariate ‘Michelson contrast of coat’ (0, 0.124, 0.330, 0.595, 0.861). This model only included random slopes over landing area.

Additionally, we used a third model (*N*=16) to analyse the effect of the size of pattern elements (experiment 4) on tabanid landings using a negative binomial distribution with log-link. Compared with the previous model, the only difference was that the main covariate Michelson contrast of coat was replaced by the continuous covariate log-transformed ‘patch size of coat’ (12.25, 194.6, 980.6, 1666.7, 1666.7, 20,000 cm^2^).

All models were fitted using weakly informative prior distributions [normal with mean=0 and s.d.=1 for intercept and coefficients, exponential (1) for standard deviations] and their performance evaluated using posterior predictive model checking, which compares model predictions with observed data to assess dispersion, zero-inflation and overall model fit. We ran 4 Markov–Chain–Monte-Carlo (MCMC) chains for each model and obtained coefficient estimates from a total of 16,000 post-warm-up samples. All model parameters reached reliable conversion indicators (Korner-Nievergelt et al., 2015): a Monte Carlo standard error smaller than 5% of the posterior s.d., an effective posterior sample size greater than 15% of the total sample size, and an 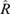 statistic value smaller than 1.01. For graphical displays of the results, we present the medians of landing rates per minute and their 95% credible intervals of the posterior distributions of fitted values for the population average, for the average video duration of 15 min pooled across the two scorers of the videos, obtained from the joint posterior distributions of the model parameters (Korner-Nievergelt et al., 2015). The same posterior distribution of fitted values was used to compute pairwise contrast ratios and their 95% credible intervals (CIs) between combinations of categorical predictor levels of interest. Their effect size is proportional to the deviation of median ratio values from 1, and the robustness of the result by the degree of overlap of the 95% CI with 1. When a continuous covariate was included in the model, we report the proportional change in number of horsefly landings per increase of one unit in the covariate of interest.

## RESULTS

### Grey, regular and irregular checked coats

**Fig. 2.**
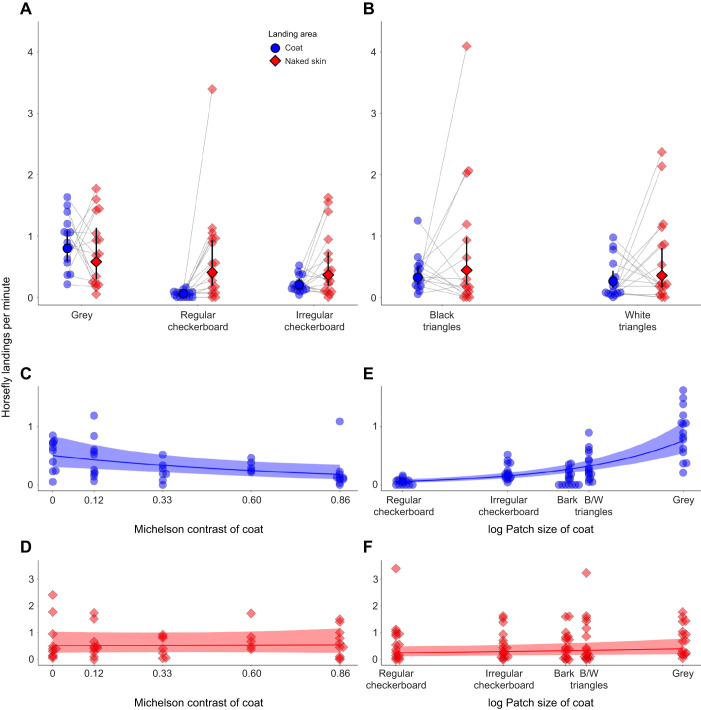
**Rate of horsefly landings as a function of coat type, Michelson contrast of coat and pattern size of coat.** Rate of horsefly landings per minute on grey checkerboard (A) and triangular (B) coat types. (C,D) Rate of horsefly landings with increasing Michelson contrast on coats (C) or on skin (D). (E,F) Rate of horsefly landings with varying patch size on coats (E) or on skin (F). Each point displays the rate of landings averaged over the two observers. Median model predictions (filled markers, dotted lines) and their 95% credible intervals (CIs; error bars, shaded areas), for the average video duration of 15 min pooled across the two scorers of the videos, are computed from the posterior distribution of fitted values obtained from the joint posterior distribution of model parameters. Group comparisons can be visually assessed by estimating the degree of overlap between the 95% CIs of each group.

Landings on the uniform grey control coat were 13 times higher than on the regular checkerboard coat and 4 times higher than on the irregular checkerboard coat (Fig. 2A, Table 1, Table S1). We also found that landings on the standard regular check patterns were 0.29 times lower compared with the irregular random check patterns; however, we observed a similar rate of landings on their naked heads, bellies or legs (Fig. 2A, Table 1).

**Table 1.**
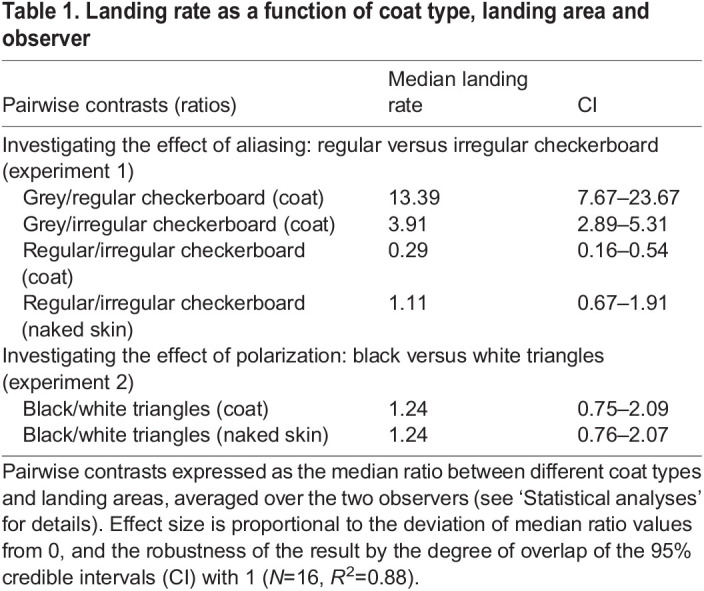
Landing rate as a function of coat type, landing area and observer

### Polarization

We found similar rate of landings between coats with black triangular patches along the dorsum compared with coats with white triangular patches along the dorsum whether we compared landings on coats or landings on naked areas of the horse (Fig. 2B, Table 1, Table S1).

### Contrast

There was a robust decrease in rates of landings on coats progressing from the uniformly grey coat that received most landings to striped coats with 0.124 through to 0.86 Michelson contrast, the last of which best mimicked a zebra's black and white stripes. For every increase of 0.01 in Michelson contrast, coat landings decreased by approximately 1.2% (95% CI: from −1.8 to −0.6%). Landing rates remained approximately stable on the naked part of the animal, however (estimated change of 0.06%, 95% CI: from −0.5 to 0.6) (Fig. 2C,D Table 2, Table S2).

**Table 2.**
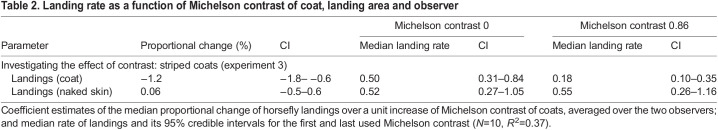
Landing rate as a function of Michelson contrast of coat, landing area and observer

### Size of pattern elements

Tabanid landings on coats increased by 41% (95% CI: from 33 to 50%) for every increase of 1 unit of the log transformed patch size (Fig. 2E, Table 3, Table S3). The increase in rates of landings on the naked parts of the animal wearing these different coats was instead only 5.8% (95% CI: from 0.8 to 11%) (Fig. 2F, Table 3, Table S3).

**Table 3.**
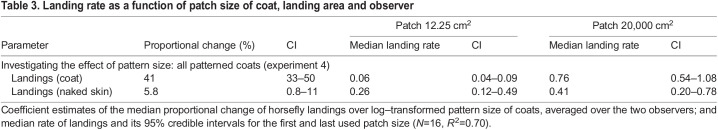
Landing rate as a function of patch size of coat, landing area and observer

## DISCUSSION

We were able to confirm one of the hypotheses proposed for zebra stripes being effective in thwarting horsefly attack and infer another hypothesis but we could find little support for the other two ideas. Our most robust result is that there is a strong effect of contrast on rates of horsefly *H. pluvialis* and *T. bromius* landings in that weakly contrasting striped coats receive considerably more landings than strongly contrasting striped coats. This finding provides an explanation for why zebras have such strikingly contrasting black and white stripes on their pelage.

There is also a suggestion that tabanid horseflies are attracted to large dark objects in their environment but less to dark broken patterns (experiment 4). Specifically, all-grey coats are associated with by far the most landings, followed by the black dorsal triangle coat, then the white triangle coat, bark and checkerboard coats in no particular order. This suggests that any ungulate that reduces the dark area of its outline against the sky will benefit in terms of reduced ectoparasite attack. It should be noted, however, that pattern shape and length of pattern edge were not controlled for on our coats, and we considered the grey coat as a single pattern element although it is not black and it has no high contrast internal pattern edges. Nevertheless, we did control for luminance with all coats being the same, and our finding is very much in line with those from several other studies that smaller pattern elements elicit fewer landings by biting flies than larger pattern elements (Brady and Shereni, 1988; Colvin and Gibson, 1992; Egri et al., 2012a).

In contrast to these positive and suggestive findings, we uncovered only weak evidence for aliasing as being a mechanism for deterring landing behaviour in tabanids. This optical illusion critically necessitates regularity in pattern in order to induce visual aliasing that disrupts the optic flow. The spatial period of the checkerboard patterns used in our experiment was 7.0 cm, which, at a viewing distance of 1 m, would correspond to an angular period of ∼4 deg. Thus, spatio-temporal aliasing could occur at this distance if the inter-ommatidial angle falls below half this value. This value of the interommatidial angle is approximately realistic for horseflies (∼1 deg; M.J.H., unpublished data). By way of comparison, human vision peaks at a foveal inter-receptor angle that is 100 times smaller than that of the horsefly (Hirsch and Curcio, 1989) and so equivalent aliasing effects could occur at much larger distances. A necessary prerequisite for aliasing is that the pattern must have regular repeating elements that may be misregistered when seen in motion. Horsefly landings occurred at a slightly higher rate on the random checkerboard (with no regularity in pattern) than on the regular check pattern which might support the aliasing hypothesis but the effect was not strong compared with the regular check pattern and this finding can instead be attributed to irregular checkerboard coats having considerably larger patch sizes owing to overlapping randomly-placed squares (95th percentile: 194.6 cm^2^), than the regular checkerboard (12.25 cm^2^). More generally, our data provide a cautionary tale for accepting perceptual illusions seen by humans (Kelley and Kelley, 2013) as explanatory factors in mechanisms underlying behaviour of non-human animals (How and Zanker, 2014).

Finally, our study finds no evidence for polarization promoting tabanid landings, at least for fragmented dark objects. Given that the degree of polarization is greater when reflected from black than from white surfaces, and that horizontal surfaces reflect horizontally polarized light more than oblique or vertical surfaces, as demonstrated by Egri and colleagues (2012b) for equids, we would expect that more flies would land on the black triangle coat. This coat had substantial amounts of black material covering the horse's neck, withers, dorsum and rump, whereas the white triangle coat consisted of white cloth in these areas. Importantly, overall, both coats were equally bright as they consisted of equal areas of white and black material. That we found no differences in landings on the two coats suggests that polarized light may not be an important factor in attracting tabanids in this context. Nonetheless, we accept that sunlight is not intense in Britain during many days of summer and any effects of polarization may be reduced under such weather conditions.

It is important to note that we could find no differences in the rate of landings on the naked areas of horses wearing different coats, indicating that, where they occurred, the effects of coats are local rather than acting at a distance from the animal. It appears flies were attracted to horses from a distance but then decided to veer away from landing on some coats but not others.

In conclusion, and in conjunction with other studies, our findings first show why zebras are striped since a sharply striped pattern receives less tabanid landings than a uniform coat of the same luminance. Other research has similarly shown that striping is more effective than an entirely white coat (Egri et al., 2012a). Second, we surmise that the size of the dark object is an important component in preventing tabanid landings, and we speculate that black stripes, by nature of being thin, serve to minimize the size of local features on an equid target that are attractive to biting flies. This result replicates previous findings showing that small-sized blobs are more effective than large blobs in deterring tabanid landings using artificial targets (Blaho et al., 2012). Third, we suggest that polarization is not a critical component of attracting horseflies and that previous empirical studies that supported this conjecture may have necessarily confounded polarization with brightness which are extremely difficult to tease apart in experimental situations. Finally, we tentatively reject aliasing as being a mechanism preventing flies from landing, and together with our previous work on failure to find evidence for an aperture effect (How et al., 2020), can dismiss disruption of optic flow as being the underlying cause of horseflies' inability to make controlled landings on zebras. Our working hypothesis now is that horseflies are attracted to equid hosts owing to a combination of odour at a distance, then size of the animal contrasted against the sky or vegetation at a middle distance. But at close range, where they can no longer see the body's outline, flies make a visual switch to local features. If these are small dark objects contrasted against a light or white background, the horsefly no longer recognizes this as a host target and veers away. The contrast of stripes and their relatively small size are therefore the key elements of how stripes operate to thwart fly landings.

## Supplementary Material

10.1242/jexbio.244778_sup1 Supplementary information
