# Supplementary material for: Why don't horseflies land on zebras?
Source: J Exp Biol. 2023 Feb 17;226(4):jeb244778. doi: 10.1242/jeb.244778 (PMC10088525; doi:10.1242/jeb.244778)
Supplement: Supplementary information [file jexbio-226-244778-s1.pdf]

Table S1. Coefficient estimates of the model investigating horsefly landings as a function of coat type, landing area and observer (see Methods). Estimates are based on a Poisson distribution with log link. When the credible intervals do not overlap with zero, the estimate has a higher probability of being non-zero.

Coefficient	Mean	M. Error	95 % CI	
			Low	High
**Population-level effects**				
Intercept *[grey, body, obs. 1]*	-0.22	0.15	-0.52	0.07
Coat *[regular checks]*	-2.57	0.32	-3.21	-1.95
Coat *[irregular checks]*	-1.42	0.19	-1.80	-1.05
Coat *[black triangles]*	-0.92	0.20	-1.32	-0.51
Coat *[white triangles]*	-0.98	0.24	-1.45	-0.51
Landing area *[body]*	-0.08	0.35	-0.79	0.61
Observer *[obs. 2]*	-0.01	0.09	-0.18	0.16
Coat *[regular checks]* x Landing area *[body]*	2.18	0.28	1.65	2.75
Coat *[irregular checks]* x Landing area *[body]*	1.05	0.20	0.66	1.44
Coat *[black triangles]* x Landing area *[body]*	0.71	0.18	0.35	1.07

Coat *[white triangles]* x Landing area *[body]*	0.61	0.18	0.26	0.96
Coat *[regular checks]* x Observer *[obs. 2]*	-0.09	0.35	-0.80	0.59
Coat *[irregular checks]* x Observer *[obs. 2]*	0.10	0.21	-0.33	0.52
Coat *[black triangles]* x Observer *[obs. 2]*	0.04	0.19	-0.33	0.41
Coat *[white triangles]* x Observer *[obs. 2]*	-0.31	0.19	-0.68	0.04
Landing area *[body]* x Observer *[obs. 2]*	-0.59	0.12	-0.84	-0.34
Coat *[reg. checks]* x L. area *[body] x* Obs. *[obs. 2]*	0.20	0.38	-0.52	0.96
Coat *[irreg. checks]* x L. area *[body] x* Obs. *[obs. 2]*	-0.39	0.27	-0.92	0.15
Coat *[black triang.]* x L. area *[body] x* Obs. *[obs. 2]*	-0.21	0.25	-0.68	0.27
Coat *[white triang.]* x L. area *[body] x* Obs. *[obs. 2]*	-0.06	0.25	-0.56	0.43
**Group-level effects**				
Sd *[Intercept]*	0.53	0.11	0.34	0.79
Sd *[regular checks]*	0.84	0.24	0.48	1.39
Sd *[irregular checks]*	0.38	0.16	0.06	0.71
Sd *[black triangles]*	0.56	0.15	0.34	0.91
Sd *[white triangles]*	0.78	0.21	0.47	1.28
Sd *[body]*	1.45	0.28	1.01	2.09

Journal of Experimental Biology • Supplementary information

Table S2. Coefficient estimates of the model investigating horsefly landings as a function of Michelson contrast of coat, landing area and observer (see Methods). Estimates are based on a negative binomial distribution with log link. When the credible intervals do not overlap with zero, the estimate has a higher probability of being non-zero.

**Coefficient**	**Mean**	**M. Error**	**95 % CI**	
			**Low**	**High**
**Population-level effects**				
Intercept *[body, obs. 1]*	-0.76	0.25	-1.24	-0.27
Michelson contrast coat	-0.01	0.004	-0.02	-0.001
Landing area *[body]*	0.26	0.38	-0.49	0.99
Observer *[obs. 2]*	0.14	0.26	-0.37	0.65
Michelson contrast coat x Landing area *[body]*	0.01	0.01	-0.003	0.02
Michelson contrast of coat x Observer *[obs. 2]*	-0.01	0.01	-0.02	0.004
Landing area *[body]* x Observer *[obs. 2]*	-0.45	0.36	-1.15	0.27
Mich. contrast coat x L. area *[body] x* Obs. *[obs. 2]*	0.01	0.01	-0.01	0.03
**Group-level effects**				
Sd *[Intercept]*	0.50	0.20	0.16	0.96
Sd *[body]*	0.97	0.32	0.46	1.73

Journal of Experimental Biology • Supplementary information

Table S3. Coefficient estimates of the model investigating horsefly landings as a function of log-transformed patch size of coat, landing area and observer (see Methods). Estimates are based on a negative binomial distribution with log link. When the credible intervals do not overlap with zero, the estimate has a higher probability of being non-zero.

**Coefficient**	**Mean**	**M. Error**	**95 % CI**	
			**Low**	**High**
**Population-level effects**				
Intercept *[body, obs. 1]*	-3.68	0.32	-4.33	-3.06
Log patch size	0.34	0.04	0.27	0.43
Landing area *[body]*	2.34	0.47	1.38	3.23
Observer *[obs. 2]*	-0.02	0.40	-0.79	0.78
Log patch size x Landing area *[body]*	-0.27	0.05	-0.37	-0.17
Log patch size x Observer *[obs. 2]*	0.002	0.05	-0.10	0.10
Landing area *[body]* x Observer *[obs. 2]*	-0.39	0.47	-1.32	0.53
Log patch size x L. area *[body]* x Obs. *[obs. 2]*	-0.04	0.07	-0.17	0.09
**Group-level effects**				
Sd *[Intercept]*	0.39	0.12	0.20	0.65
Sd *[body]*	1.42	0.29	0.96	2.10

Journal of Experimental Biology • Supplementary information

Table S4. Raw data to replicate analyses investigating 1) aliasing and 2) polarization.

Click here to download Table S4

Table S5. Raw data to replicate analyses investigating 3) contrast.

Click here to download Table S5

Table S6. Raw data to replicate analyses investigating 4) pattern size.

Click here to download Table S6

Journal of Experimental Biology • Supplementary information
